# The Poor and Their Doctors

**Published:** 1888-02-04

**Authors:** 


### The Hospital

A WEEKLY INSTITUTIONAL JOURNAL OF

Science, Medicine, and Philanthropy.

*For Hospitals, Asylums, Medical Practitioners, Students, Nurses, Families, Parishes, Congregations, and the Charitable Public.*

VOL. III. No. 71.] [FEBRUARY 4, 1888.

### THE POOR AND THEIR DOCTORS.

THE doctoring of the poor and of those who are but one remove from poverty has long been a burning question both at hospitals and among private practitioners. A variety of methods have been tried, each intended to secure the best medical skill of the time for the working classes without injury to those who practise medicine among them. Clubs, provident dispensaries, private dispensaries, and out-patient departments at general hospitals, are all examples of the efforts which have been made; and it may be affirmed generally that no one of them nor all of them combined, satisfy the equation exactly. A new departure is proposed in the shape of a suggestion to turn the out-patient departments of the general hospitals into provident dispensaries; for that is really the result which the adoption of recent proposals would lead to. The subject is very old, but also very new, being indeed in such a condition of active fermentation that it cannot be considered other than as demanding the careful attention of all concerned.

The industrious poor must in times of sickness be attended by competent doctors. On that point all classes are agreed. The humanitarian holds it as an article of faith because he *is* a humanitarian; the ratepayer because the disablement of the worker means the discontinuance of his wages and the support of his family from the rates; the employer of labour because his plans are upset by the absence of the sick man, and his operations are thereby rendered more difficult and less successful. Looked at from any standpoint it is a proposition requiring no discussion, that the disabled worker must have prompt and efficient medical treatment.

But—and here lies the difficulty—where and how is he to get it? "As other people do," answers the unwary outsider; "by employing the best doctor within reach of his house and of his means." Exactly. That is quite the natural thing to think of doing! But is it also the practicable thing to do? Civilization makes many naturally simple things complex. It is easy to say that Spayde, the sexton, who is recovering from pneumonia, is to have a pint of champagne every day, but how is he to get it when both the vicar and himself are deprived of their Sunday's roast beef because the farmers refuse to pay tithes? The honest and independent-minded artizan would undoubtedly be glad to have the best medical attendance to be got near his own home, provided he could pay for it—but can he? Attendance at home for poor men who are confined to bed is generally impracticable, and, for a variety of reasons, undesirable. The patient who is so ill as to require to be *taken into the hospital* for a time is, by common consent, in his right place when he is located in the ward. Nobody doubts this, and nobody grudges him the gratuitous treatment he there obtains, least of all the private medical practitioner.

The out-patient is the shoe that pinches the favourite corns, both of hospital officials and private medical practitioners. He is always present, like the wart on Cromwell's nose, and always unsatisfactory. In many cases there is little or nothing the matter with him. Very frequently he is not even poor, but only mean—too mean to pay a doctor for himself. Not seldom his illness is of such a kind as to be discreditable to him, and no more than the just reward of his demerit. But with all his faults, virtues, and other peculiarities there he is in the out-patient department, and there he is likely to remain, a doubtful mixture, unless some winnowing fan can be discovered which shall separate once and for ever the wheat from the chaff.

Why do so many people seek the out-patient department at the neighbouring hospital? One answer is, Because they get more skilful treatment there than they can obtain at the hands of private practitioners. Is that really the case? We take leave very emphatically to doubt it. The people who seek the out-patients' room are, as is well-known, generally those who are suffering from minor ailments. Now will it be affirmed that the average practitioner is not capable of treating the minor ailments of working men and women? If he is not, What is he capable of treating? Who, we ask, treats the minor, and, indeed, the major ailments also, of the middle and upper classes? Is it not the ordinary family practitioner? And in what way is the practitioner in a wealthy district superior in professional status to him of the poorer locality. In very many cases he is not superior at all except in the power to purchase his entrance into a better kind of practice and to hold his own among a wealthier class of people. But that does not constitute superior medical knowledge and skill. If the average general practitioner is competent to treat the minor and major ailments of the middle and upper classes, so also is his brother who works in a poorer district fit to treat all kinds of cases that come in his way. So far then as the argument about the necessity for superior skill is concerned the ordinary denizen of the out-patients' room has not a leg to stand upon.

We have no desire whatever to press hardly upon the working man, but *à priori* there is no more reason why a charitable organisation should provide him with gratuitous medical attendance when he is not entirely disabled, than that a similar organisation should provide him with cheap beef, cheap tobacco, or cheap boots. In the country the average workman has no out-patient department to go to; and he manages to secure very efficient medical attendance by means of his benefit society. He does not appear to be any the worse, but rather the better, for the self-denial he is thus called upon to practise.

Instead of turning the out-patient department into a provident dispensary, our view would rather be that an effort should be made to pass every patient through the hands of the general practitioner, and that he should act as a winnowing fan for the hospitals. Let no one make an outcry against this on account of the supposed greed of the practitioner. There is no necessity for it. The general practitioner does not want people who cannot afford to pay him, and he is only too glad to send them off to the hospital at the earliest possible moment. A scheme of affiliation, whereby every duly-qualified and established medical man within a given radius of a central hospital should be attached as an out-patient physician to that hospital, seems to us eminently practicable. The doctor would not attend at the hospital, but would have fixed hours for seeing the poorer class of patients at his own house. Those who could pay a sum sufficient to save him from loss he would retain. Those who could not pay sufficient he would pass on to the hospital. Those whom his skill availed to cure he would cure; those who did not yield to treatment in a given time, he would also transfer to the out-patient department. A system of inspection might be adopted whereby a competent physician representing the hospital could at any time call upon the affiliated doctors and examine their methods of working. Or such an officer might pay periodical visits at fixed times; and such times being known, all the difficult cases could be brought for special consultation on the day of his visit.

This scheme, it seems to us, possesses many advantages. It would keep the doctors "in touch" with the hospitals; it would greatly diminish the number of their out-patients, and relieve the strain upon both their financial and their professional resources; it would put an end to the injurious competition which exists between medical men and medical charities; and it would secure to the poor equal skill and more care, besides promoting their independence and self-respect. As a contribution to the perennial and vexatious out-patient question we commend this brief outline to the sober consideration of all hospital supporters and officials. That something needs tobe done no one will deny; that that something should be just to medical practitioners, as well as efficient for the patients, is imperative if a permanent solution of the problem is to be attained.

